# DNA replication: archaeal oriGINS

**DOI:** 10.1186/1741-7007-9-36

**Published:** 2011-05-31

**Authors:** Stephen D Bell

**Affiliations:** 1 Sir William Dunn School of Pathology, South Parks Road, Oxford OX1 3RE, UK

## Abstract

GINS is an essential eukaryotic DNA replication factor that is found in a simplified form in Archaea. A new study in this issue of *BMC Biology *reveals the first structure of the archaeal GINS complex. The structure reveals the anticipated similarity to the previously determined eukaryotic complex but also has some intriguing differences in the relative disposition of subunit domains.

**See research article**: http://www.biomedcentral.com/1741-7007/9/28

## Commentary

GINS was first identified by independent genetic screens in *Saccharomyces cerevisiae*. It is a DNA polymerase accessory factor that, with Cdc45, binds to and activates MCM helicase. The factor is composed of four distinct but related subunits, Sld5, Psf1, Psf2 and Psf3, that are conserved across the eukaryotic domain of life. The name GINS arises from the first letters of the Japanese names for the numerals in the subunits: Go, Ichi, Ni and San. The sequence relationship of the four subunits suggests that they arose from a common ancestor (reviewed in [1,2]. Indeed, present day archaea, which possess a simplified form of the eukaryotic replication machinery, presumably reflective of a more ancestral state, have a simplified GINS factor. This can either be an α_2_β_2 _tetramer - containing two copies each of subunits termed Gins15 and Gins23, related to Psf1 and Sld5 and to Psf2 and Psf3 respectively, or, in a few cases, a simpler homotetrameric form, such as in *Thermoplasma acidophilum*.

In eukaryotes, GINS, in conjunction with Cdc45 and additional factors, is recruited to the replicative helicase MCM(2-7) at replication origins prior to the initiation of DNA replication. When MCM(2-7) leaves the replication origin and drives replication fork movement, GINS and Cdc45 travel with it, along with an array of additional factors, in what has been termed the replisome progression complex [3]. However, the precise role of GINS in this higher order complex remains poorly understood. Interestingly, the Cdc45-MCM-GINS (CMG) sub-complex appears to be a highly stable assembly and has been demonstrated to have robust DNA helicase activity *in vitro *[4].

## Variability among GINS complexes from different life domains

The GINS subunits show a permutation of their domain organization - structural studies of Psf1 and Sld5, the human homologs of archaeal Gins15, show that they have an amino-terminal α-helix domain (A) and a carboxy-terminal β-strand-rich domain (B). Homologous domains are found in Psf2 and Psf3, the human homologs of archaeal Gins23, but in a permuted, BA, order, with the β-sheet preceding the α-helical domain [1]. Similarly, the current work by Oyama and colleagues reveals that the individual Gins23 and Gins15 subunits of the GINS complex from the archaeon *Thermococcus kodakaraensis *have an analogous permutation, with Gins15 being AB and Gins23 being BA.

Overall, the archaeal complex, despite having a simplified subunit composition relative to humans, has striking similarity to the human GINS assembly, although there are some differences in the contacts observed between Gins15 and Gins23 from those seen between their eukaryotic counterparts. Probably the most significant difference lies in the positioning of the carboxy-terminal β-strand-rich B-domain of Gins15 when compared with the analogous features of its human counterparts Psf1 and Sld5. Despite the conservation of Sld5 and Psf1, the B-domain of human Sld5 is involved in contacts with Psf2, while the B-domain of Psf1 is highly mobile and dispensable for GINS complex formation. In this regard, Gins15 is more reminiscent of Psf1 than Sld5, as the B-domain is not required for formation of the archaeal GINS complex [5]. Thus, while the presence of GINS is conserved from archaea to humans, the subunit composition and details of the geometry of the assembly vary.

## Functional significance of structural differences

What then are the implications of this new archaeal structure for the formation and organization of the higher order macromolecular assembly at the replication fork? To address this, the new structure must be viewed in the context of two further recent papers and some older data from the archaeon *Sulfolobus solfataricus*. The first study is the identification by Kelman and colleagues of a RecJ family nuclease, GAN, that interacts specifically with Gins15 in *Thermococcus kodakaraensis *[6]. This is highly reminiscent of the situation in another archaeon, *Sulfolobus solfataricus*, where the GINS complex co-purifies with a further protein, RecJdbh, named for its significant homology to the single strand DNA binding domain of RecJ [7]. Binding studies revealed that, as with GAN, RecJdbh interacts specifically with Gins15. Although the precise interaction interface has yet to be mapped, given that the B-domain of Gins15 appears mobile and suitably exposed, it is highly tempting to speculate that this domain of Gins15 is responsible for the interaction with GAN and RecJdbh. As in eukaryotes, archaeal GINS has been shown to interact physically with MCM. Studies in *Sulfolobus *revealed that this interaction is mediated by Gins23. Furthermore, in *Sulfolobus*, Gins23 also interacts with the DNA primase [7]. Perhaps the single-stranded DNA binding activity of the RecJdbh (and by analogy GAN) plays a role in directing single-stranded DNA generated by the helicase action of MCM to the catalytic site of primase, ensuring coupling of DNA unwinding and priming activity. In this context, it is particularly exciting to note that Kelman and colleagues comment on a potential similarity between the predicted structure of eukaryotic Cdc45 and RecJ [6]. Thus, the archaeal RecJ-like proteins may serve as analogs or even homologs of eukaryotic Cdc45.

**Figure 1:**
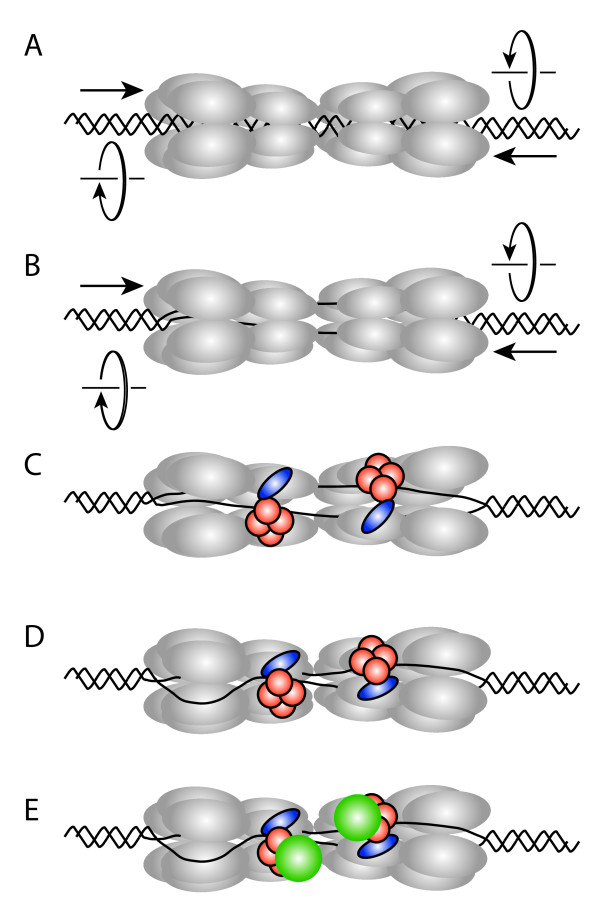
**Model for the initial assembly of the archaeal replisome based on recent advances in the eukaryotic DNA replication field (see **[8,9]**)**. **(a) **A double hexamer of MCM (gray) is loaded on double-stranded DNA at an archaeal replication origin. **(b) **The two individual hexamers are held together, so that, instead of moving apart, they will pump DNA into the central cavity of the assembly. If the pumping has a defined handedness, DNA will be unwound in the centre of the double hexamer. **(c) **The GINS complex (orange) in conjunction with RecJdbh or GAN (blue) stabilizes an open form of the hexameric MCM and allows extrusion of one DNA strand. **(d) **Resealing the MCM hexamer traps the displaced strand between the outside of MCM and the GINS assembly. **(e) **GINS recruits DNA primase (green).

A recent single particle EM reconstruction study by Berger, Botchan and colleagues has revealed the architecture of the eukaryotic Cdc45-MCM(2-7)-GINS complex [8]. The MCM(2-7) complex is shown to form an open ring with a gap between subunits MCM2 and MCM5. Importantly the GINS and Cdc45 proteins bridge across this gap (Figure 1c). In the presence of a non-hydrolyzable analog of ATP, the gate in MCM shuts, forming a dual pore structure, one pore through the centre of the core MCM(2-7) and another formed between GINS/Cdc45 and the outer surface of the MCM (Figure 1). While the fine details of the interactions between GINS and Cdc45 remain to be resolved, it is possibly significant that while the flexible B-domain of Psf1 is not required for GINS complex assembly (mirroring the case with the B-domain of Gins15 in archaea), it is required for formation of the higher order Cdc45-MCM-GINS complex [8].

What is the significance of this dual pore structure of eukaryotic CMG? All available data in the eukaryotic system indicate that MCM is loaded onto double stranded DNA at replication origins as a head-to head double hexamer [9]. If the two hexamers interact with one another, then as they attempt to translocate in opposite directions they will instead pump DNA into the centre of the assembly (Figure 1b). With appropriate rotational stress the DNA may begin to unwind. GINS/Cdc45, by stabilizing an open form of the MCM ring, could allow extrusion of a single strand (Figure 1c). Resealing of the MCM ring would then generate a structure with a single strand of DNA passing through the centre of the helicase and the second displaced strand trapped by the outer pore generated by MCM, Cdc45 and GINS (Figure 1d). Fluorescence resonance energy transfer experiments with archaeal MCM have revealed that the strand passing through the center of the helicase would be the leading strand template, while the displaced strand would be the lagging strand template [10]. Thus, if such a double pore structure also exists in archaea (with RecJdbh/GAN taking the place of Cdc45) then the resultant assembly would deliver the lagging strand template directly to the DNA primase (which interacts with Gins23) (Figure 1e). Interestingly, it has been proposed that human GINS can also functionally interact with human DNA primase, suggesting a conservation of this coupling throughout evolution.

The structure of archaeal GINS complex represents a first step towards understanding the architecture of the replication fork assembly - clear future goals lie in structural analyses of higher order assemblies coupled with detailed biochemical investigations of the interactions between replisome components and their consequence for the coordination of DNA unwinding, priming events and subsequent DNA synthesis at the replication fork.
